# Extra Limites

**Published:** 1821-12-01

**Authors:** Henry Reeder

**Affiliations:** 42, St. Thomas's Street, Southwark


### EXTRA LIMITES.

### *Dr. Reeder's Reply to the Review of his Work in the 6th Number of this Journal.*

---

I think it proper to observe, regarding what was advanced relative to my Treatise on Diseases of the Heart in the last number of the Medico-Chirurgical Journal, that I consider the review of it imperfect, the analytical account garbled, and the critical remarks illiberal and unjustifiable in their whole tenour. It will obviously appear to every one who has merely glanced over the work, that the review of it is extremely imperfect: in it, for example, there are first a few vague observations on that part of the performance which treats on Inflammation of the Heart; next occur a few cursory remarks on Angina Pectoris, but no mention whatever is made of the views I have taken of that disease, in regard to its various causes, diagnosis, nature, &c. which altogether are very different from those of any preceding writer. The other divisions of the work are also passed over in a manner equally slight. The analytical account, too, is entirely garbled, inasmuch as the annotations are made upon the most unimportant parts of the treatise, and are also calculated to misrepresent. And the illiberal nature of the critical remarks is evinced from beginning to end, in proof of which it may suffice to mention that *because a little* h *for a great one, and a small* g *for a large one,* (in situations of no importance, and in which their use is sanctioned by the authority of many of the *best* modern writers) were not considered typographical errors sufficiently momentous to claim a place in the table of errata, yet they must be deemed worthy of two distinct commentaries not less disingenuous, than sarcastic. In the review, moreover, scarcely one word is mentioned respecting the diagnostic part of the work, which sets forth the methods by which the various diseases of the heart may be discriminated, and especially affections of an organic from those of a sympathetic nature, and all which is original, for such I believe had never been attempted in a similar manner. I am perfectly indifferent as to what term the reviewers may bestow upon the volume, whether that of "compilation" or any other name; yet it is such an one as contains a fair proportion of original matter, deduced from personal observation. I am willing to believe, too, that it comprizes a larger number of facts in less compass, or in a more condensed and methodical form, than most books that have been published on Diseases of the Heart; and that it comprehends what has been made known, on that subject, in this country, and also a considerable proportion of the really useful information which has been published on the Continent. It is true, I have not taken any notice of the stethescope, invented by Laennec, nor of the inane wonders that have been achieved by it; —an instrument calculated perhaps to amuse, for a short time, those who pursue novelty with such extraordinary avidity; but which, with the more sober-minded, is, I am disposed to surmise, destined, ere long, to pass through the ordeal of experiment into oblivion.

HENRY REEDER.

*42, St. Thomas's Street, Southwark.*

---

☞ There is room for comment on Dr. Reeder's remonstrance; but we hold it very ungenerous to counteract the effect of an author's reply by any observations at the time the reply is published, unless the subject matter requires immediate notice.—We hope Dr. Reeder will be cooler after this evolution of caloric.

